# Association between GDF5 rs143383 genetic polymorphism and musculoskeletal degenerative diseases susceptibility: a meta-analysis

**DOI:** 10.1186/s12881-018-0685-7

**Published:** 2018-09-14

**Authors:** Xin Huang, Weiyue Zhang, Zengwu Shao

**Affiliations:** 1 Department of Orthopaedics, Union Hospital, Tongji Medical College, Huazhong University of Science and Technology, Wuhan, 430022 China; 2 Department of Endocrinology, Union Hospital, Tongji Medical College, Huazhong University of Science and Technology, Wuhan, 430022 China

**Keywords:** GDF5 gene, Polymorphisms, Musculoskeletal degenerative diseases, Meta-analysis

## Abstract

**Background:**

Several studies have assessed the association between GDF5 rs143383 polymorphism and the susceptibility of musculoskeletal degenerative diseases, such as intervertebral disc degeneration (IDD) and osteoarthritis (OA), but the results are inconsistent. The aim of our study was to evaluate the association between them comprehensively.

**Methods:**

A systematical search was conducted on PubMed, Scopus, Web of Science (WOS), Embase, and the Cochrane Library databases updated to April 20, 2018. Eligible studies about polymorphisms in GDF5 gene and risk of IDD or OA were included. Pooled odds ratios (ORs) and 95% confidence intervals (95% CIs) were utilized.

**Results:**

Fifteen studies with a total of 5915 cases and 12,252 controls were finally included in our study. Meta-analysis of GDF5 rs143383 polymorphism was statistically associated with increased risk of musculoskeletal degenerative diseases under each genetic model (allele model: OR = 1.32, 95% CI 1.19–1.48, *P* = 0.000; homozygote model: OR = 1.80, 95%CI 1.49–2.16, *P* = 0.000; heterozygote model: OR = 1.37, 95%CI 1.21–1.55, P = 0.000; dominant model: OR = 1.56, 95%CI 1.39–1.75, *P* = 0.000; recessive model: OR = 1.39, 95%CI 1.20–1.60, *P* = 0.000). Stratified analyses based on disease type showed a significant association between the GDF5 rs143383 polymorphism and increased risk of IDD and OA under all genetic models studied. When stratified with ethnicity, pooled outcomes revealed that this polymorphism was significantly related with increased risk of musculoskeletal degenerative diseases in both Asian and Caucasian populations under all genetic models studied.

**Conclusions:**

The present study suggested that GDF5 rs143383 polymorphism was significantly associated with susceptibility to musculoskeletal degenerative diseases.

**Electronic supplementary material:**

The online version of this article (10.1186/s12881-018-0685-7) contains supplementary material, which is available to authorized users.

## Background

Intervertebral disc degeneration (IDD) and osteoarthritis (OA) are two major musculoskeletal degenerative diseases that bring about pain, physical limitations and disability of patients. IDD has been one of the important causes to low back pain (LBP) and motor deficiency. Lumbar disc herniation (LDH) is caused mainly by IDD because the degeneration and herniation of nucleus pulposus exist in the lumbar intervertebral disc [1]. OA is a chronic age-associated disease resulted from articular cartilage degeneration [2, 3], which has a profound influence on the functioning of synovial joints, primarily the knee, hip, and hands [3]. Apart from aging, hormonal, environmental and behavioral factors, genetic factor has been implicated in the etiology and pathogenesis of musculoskeletal degenerative diseases [4–6].

Growth differentiation factor 5 (GDF5) is a member of the transforming growth factor-β (TGF-β) superfamily with high articular cartilage specificity [7]. Studies have revealed the significant value of GDF5 gene in musculoskeletal processes including endochondral ossification, synovial joint formation, tendon repair and bone production [8–10]. It is also suggested that GDF5 is effective in enhancing the proliferation and matrix anabolism of intervertebral disc cells [11–13]. The + 104 T/C polymorphism (rs143383) in the 5′-untranslated region (UTR) of GDF5 gene influences transcriptional activity in the gene core promoter, and lower GDF5 expression has been detected in individuals carrying T alleles.

Although several meta-analyses have revealed a possible relationship between the GDF5 rs143383 and knee OA and other common phenotypes OA [6, 14–16], several new studies have also reported an association between rs143383 and the risk of IDD [17–19] and other phenotypes of OA [5, 20–23]. Therefore, the data needs to be updated and more reliable studies are warranted to conclude whether the association varies by disease type and ethnicity. Our study conducted a meta-analysis to shed some light on the relationship between GDF5 rs143383 polymorphism and the susceptibility of IDD and OA using all published case–control association studies.

## Methods

### Search strategy

A computerized literature search was conducted in the PubMed, Scopus, Web of Science (WOS), Embase, and the Cochrane Library databases up to April 20, 2018. The search method of our study followed the terms such as: (“IDD” or “LDD” or “LDH” or “LBP” or “Intervertebral Disc Degeneration” or “OA” or “osteoarthritis”) and (“GDF5” or “rs143383” or “GDF5 + 104 T/C”) and (“polymorphisms” or “variants” or “variation” or “SNP”). Eligible articles that matched the inclusion criteria were included. Moreover, the references of articles were examined one by one to avoid missing any eligible studies. When the important data were not available, we tried to contact researchers of some articles.

### Inclusion and exclusion criteria

A study that is eligible for inclusion must meet the following criteria: (1) case–control study or cohort study including both case and control groups, (2) detection of GDF5 polymorphisms and IDD or OA risk, (3) having an accessible genotype frequency for calculating an odds ratio (OR) or hazard ratio (HR) with 95% confidence interval (95% CI), (4) genotype frequencies in controls must conform to Hardy-Weinberg equilibrium (HWE). Whereas, reviews, case reports or serious, or similar works were all eliminated. We also eliminated the studies with genotype frequencies not in HWE [24].

### Data extraction and quality assessment

Two investigators (Xin Huang, Weiyue Zhang) were assigned to assess the eligibility of all studies. And the relevant data for analysis were extracted on their own. Moreover, a third investigator (Zengwu Shao) resolved the disagreements when necessary. The important data were collected as follows: name of first author, year, countries, ethnicity, sample size, disease, sex, age, genotyping methods, and allele frequencies of GDF5 rs143383. The study quality was assessed in accordance with the Newcastle-Ottawa Scale (NOS). The study was considered high quality with the scores were ≥ 7.

### Statistical analysis

The statistical data was analyzed by Stata version 14.0. Outcomes were calculated by odds ratios (ORs) and 95% confidence intervals (CIs). Genotype frequencies of GDF5 rs143383 polymorphism for HWE were calculated using the chi-square test, and *P* < 0.05 was regarded as significant disequilibrium. The chi-square test and the I^2^ statistic were utilized to assess the between-study heterogeneity. If an I^2^ value of < 50%, it was considered that no significant heterogeneity existed [25]. A random effects model was utilized when there was a significant heterogeneity. On the contrary, the fixed effects model was utilized. Moreover, we further made subgroup analyses to evaluate the source of heterogeneity. Begg’s and Egger’s methods were mainly utilized to assess publication bias. And sensitivity analyses were to evaluate the stability of major outcomes and possible source of heterogeneity.

## Results

### Search results

**Fig. 1:**
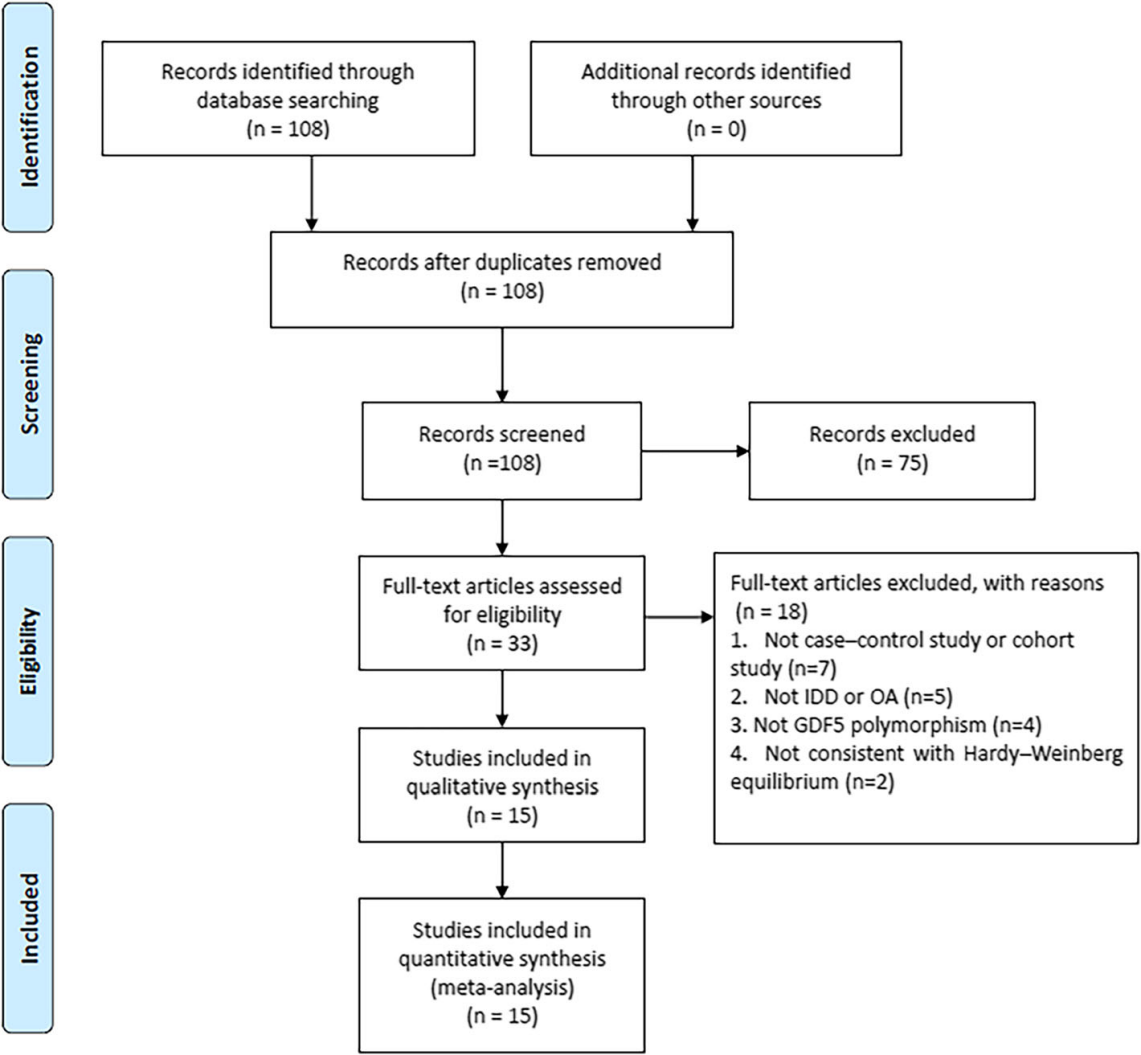
Flowchart of the study selection process

The study search is shown in the flow diagram (Fig. 1). 108 relevant articles were collected during the databases search. Furthermore, 75 were eliminated during abstract review, and 33 for further review. During the full-text review, 18 articles were eliminated for the following reasons: seven were neither case–control study or cohort study, five were not associated with IDD or OA, four were not GDF5 polymorphisms on IDD or OA risk, two was not consistent with HWE. To sum up, 15 studies with 5915 cases and 12,252 controls were included in the present study.

### Study selection and characteristics

The main features of each eligible study are summarized in details (Table 1). Among these eligible studies, three articles investigated value of GDF5 rs143383 in IDD risk in seven independent populations. In addition, twelve articles examined effects of GDF5 rs143383 on the risk of OA in fifteen independent populations. Ten in the included studies had been made among Asian populations, and twelve were in Caucasian populations. The years for publication ranged from 2007 to 2017. In all these articles, genotype frequencies in controls conformed to HWE (Table 2). According to NOS, the quality scores of all eligible articles ranged from 7 to 8, which indicated a good quality (Additional file 1: Table S1). Furthermore, the definitions of diseases, inclusions, and exclusions of patients in each study are also shown (Additional file 1: Table S2).

**Table 1:** Main characteristics of the studies included in this meta-analysis

First author	Year	Country	Ethnicity	Sample size		Disease	Sex	Age (y)	Genotyping method	Quality	Citation
				Case	Control						
Mu J	2014	China	Asian	231	370	LDH	Both	21.9	DNA analyzers	Y	[17]
Mu J	2013	China	Asian	305	587	LBP	Both	48.4	DNA analyzers	Y	[18]
Williams FMK ^a^	2011	UK	Caucasian	194	1268	LDD	Both	65.7	KASPar chemistry	Y	[19]
Williams FMK ^b^	2011	UK	Caucasian	33	539	LDD	Both	54.7	KASPar chemistry	Y	[19]
Williams FMK ^c^	2011	UK	Caucasian	54	704	LDD	Both	62.9	KASPar chemistry	Y	[19]
Williams FMK ^d^	2011	UK	Caucasian	18	574	LDD	Both	53.6	KASPar chemistry	Y	[19]
Williams FMK ^e^	2011	UK	Caucasian	14	116	LDD	Both	65.8	KASPar chemistry	Y	[19]
Tülüce Y	2017	Turkey	Caucasian	95	77	OA	Both	62.5	PCR-RFLP	Y	[20]
Abd Elazeem MI	2017	Egypt	Caucasian	50	50	Primary knee OA	Both	56.5	TaqMan	Y	[21]
Sabah-Ozcan S	2016	Turkey	Caucasian	94	279	Knee OA	Both	58.4	PCR-RFLP	Y	[22]
Xiao JL	2015	China	Asian	114	126	Temporomandibular joint OA	Both	33.6	PCR-RFLP	Y	[23]
Mishra A	2013	India	Asian	300	300	Knee OA	Both	54.0	PCR-RFLP	Y	[5]
Tawonsawatruk T	2011	Thailand	Asian	90	103	Knee OA	Both	68.5	PCR-RFLP	Y	[29]
Cao Z	2010	Korea	Asian	276	298	Knee OA	Both	63.0	PCR-RFLP	Y	[30]
Valdes AM	2009	UK	Caucasian	259	509	Knee OA	Both	68.5	Allele-specific PCR	Y	[31]
Tsezou A	2007	Greece	Caucasian	251	268	Knee OA	Both	67.9	Direct sequence	Y	[32]
Miyamoto Y ^a^	2007	Japan	Asian	718	861	Knee OA	Both	71.9	TaqMan	Y	[33]
Miyamoto Y ^b^	2007	China	Asian	313	485	Knee OA	Both	58.8	TaqMan	Y	[33]
Miyamoto Y ^c^	2007	Japan	Asian	998	983	Hip OA	Both	71.9	TaqMan	Y	[33]
Southam L ^a^	2007	UK	Caucasian	509	822	Knee OA	Both	65.0	PCR-RFLP	Y	[34]
Southam L ^b^	2007	Spain	Caucasian	274	1196	Knee OA	Both	65.0	TaqMan	Y	[34]
Shin MH	2012	Korea	Asian	725	1737	Knee OA	Both	67.4	High resolution melting analysis	Y	[35]

^a,b,c,d^and ^e^ denote an independent study in one article, respectively; *LDD* lumbar disc degeneration, *LDH* lumbar disc herniation, *LBP* low-back pain, *OA* osteoarthritis, *RT-PCR* real-time polymerase chain reaction, *PCR-RFLP* polymerase chain reaction-restriction fragment-length polymorphism, *Y* yes

**Table 2:** Genotype distribution of the studies included in this meta-analysis

Study ID	Year	Ethnicity	Disease	Case group			Control group			P for HWE
GDF5 rs143383				CC	CT	TT	CC	CT	TT	
Mu J	2014	Asian	LDH	8	79	144	39	158	173	0.743
Mu J	2013	Asian	LBP	10	89	206	58	254	275	0.953
Williams FMK ^a^	2011	Caucasian	LDD	21	103	70	218	586	464	0.159
Williams FMK ^b^	2011	Caucasian	LDD	4	14	15	94	252	193	0.453
Williams FMK ^c^	2011	Caucasian	LDD	6	23	25	119	312	273	0.067
Williams FMK ^d^	2011	Caucasian	LDD	2	7	9	72	256	246	0.671
Williams FMK ^e^	2011	Caucasian	LDD	1	8	5	16	42	58	0.073
Tülüce Y	2017	Caucasian	OA	24	39	32	8	39	30	0.366
Abd Elazeem MI	2017	Caucasian	OA	14	16	20	13	25	12	0.998
Sabah-Ozcan S	2016	Caucasian	OA	14	43	37	52	153	74	0.083
Xiao JL	2015	Asian	OA	5	47	62	19	54	53	0.396
Mishra	2013	Asian	OA	46	130	124	56	160	84	0.188
Tawonsawatruk T	2011	Asian	OA	11	41	38	23	47	33	0.424
Cao Z	2010	Asian	OA	11	115	150	26	113	159	0.397
Valdes AM	2009	Caucasian	OA	35	98	126	84	244	181	0.908
Tsezou A	2007	Caucasian	OA	30	126	95	44	125	99	0.669
Miyamoto Y ^a^	2007	Asian	OA	31	243	444	58	330	473	0.966
Miyamoto Y ^b^	2007	Asian	OA	19	97	197	48	193	244	0.283
Miyamoto Y ^c^	2007	Asian	OA	31	266	701	70	371	542	0.552
Southam L ^a^	2007	Caucasian	OA	52	238	219	126	372	324	0.262
Southam L ^b^	2007	Caucasian	OA	36	136	102	194	563	439	0.549
Shin MH	2012	Asian	OA	38	305	382	106	689	942	0.176

^a,b,c,d^ and ^e^ denote an independent study in one article, respectively, *HWE* Hardy-Weinberg equilibrium, *LDD* lumbar disc degeneration, *LDH* lumbar disc herniation, *LBP* low-back pain, *OA* osteoarthritis

### Association between GDF5 rs143383 and musculoskeletal degenerative diseases

**Fig. 2:**
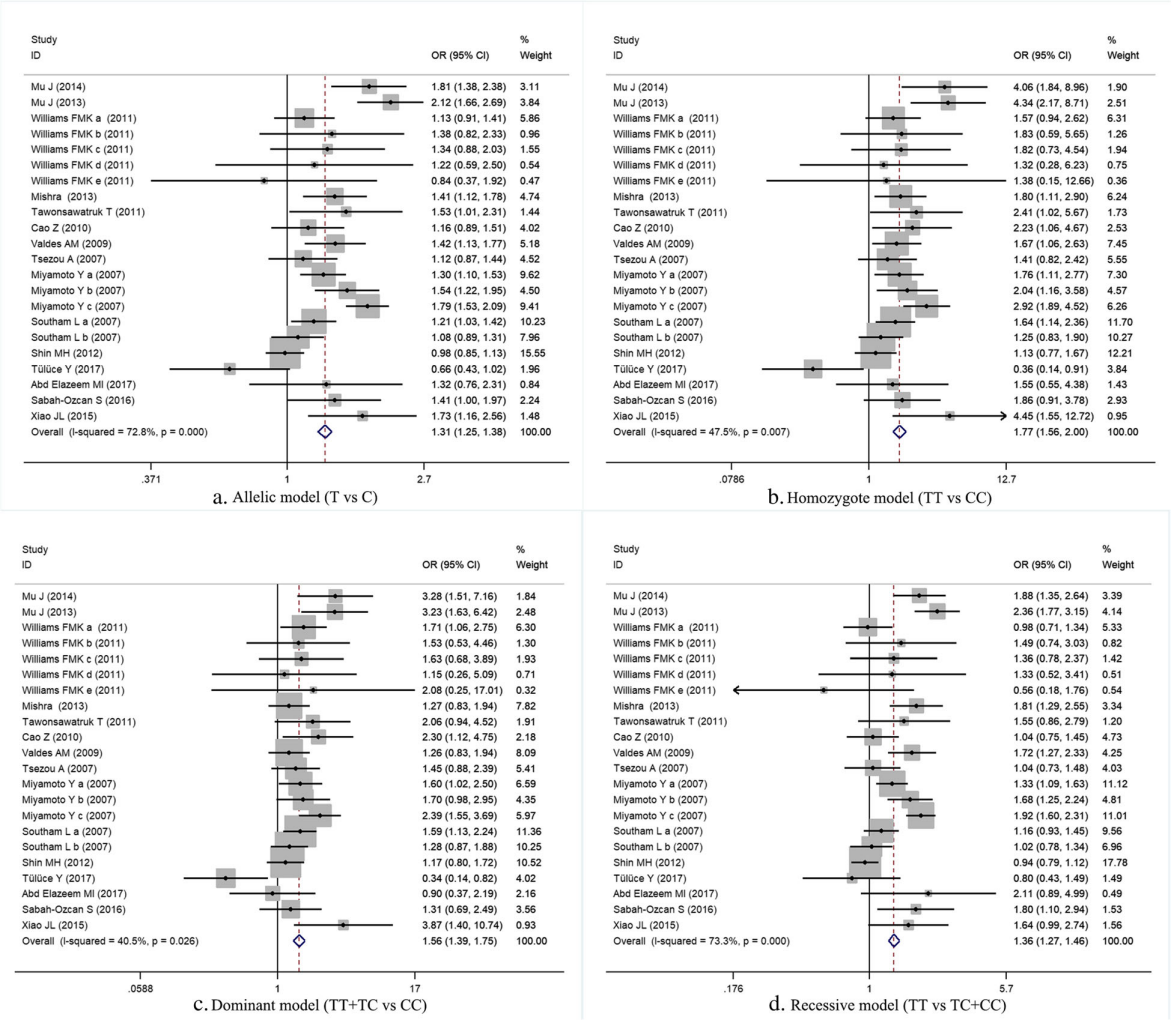
(**a**) Meta-analysis for GDF5 rs143383 polymorphism under allele model; (**b**) Meta-analysis for GDF5 rs143383 polymorphism under homozygote model; (**c**) Meta-analysis for GDF5 rs143383 polymorphism under dominant model; (**d**) Meta-analysis for GDF5 rs143383 polymorphism under recessive model

A total of 5915 patients and 12,252 controls were included in our study on GDF5 rs143383 polymorphism. There was a significant relationship between GDF5 rs143383 polymorphism and increased risk of musculoskeletal degenerative diseases under each genetic model (allele model: OR = 1.32, 95% CI 1.19–1.48, *P* = 0.000; homozygote model: OR = 1.80, 95%CI 1.49–2.16, *P* = 0.000; heterozygote model: OR = 1.37, 95%CI 1.21–1.55, *P* = 0.000; dominant model: OR = 1.56, 95%CI 1.39–1.75, *P* = 0.000; recessive model: OR = 1.39, 95%CI 1.20–1.60, *P* = 0.000) (Fig. 2 and Table 3). The heterogeneity of studies on this polymorphism was< 50%, under homozygote, heterozygote and dominant models.

**Table 3:** Meta-analysis of the association between GDF5 rs143383 and musculoskeletal degenerative diseases

GDF5 rs143383	Subgroup	Assessment of association			Assessment of heterogeneity		
		OR	95% CI	P	Pooling model	I^2^ (%)	*P*
Allelic model	Overall	1.32	1.19–1.48	0.000	Random	72.8	0.000
	Disease						
	IDD	1.45	1.13–1.86	0.004	Random	67.6	0.005
	OA	1.28	1.14–1.44	0.000	Random	73.4	0.000
	Ethnicity						
	Asian	1.49	1.25–1.77	0.000	Random	83.2	0.000
	Caucasian	1.18	1.09–1.28	0.000	Fixed	17.1	0.276
Homozygote model	Overall	1.80	1.49–2.16	0.000	Fixed	47.5	0.007
	Disease						
	IDD	2.33	1.55–3.51	0.000	Fixed	29.5	0.203
	OA	1.67	1.36–2.05	0.000	Random	50.1	0.014
	Ethnicity						
	Asian	2.28	1.71–3.05	0.000	Random	58.9	0.009
	Caucasian	1.46	1.23–1.74	0.000	Fixed	0.0	0.460
Heterozygote model	Overall	1.37	1.21–1.55	0.000	Fixed	26.7	0.122
	Disease						
	IDD	1.84	1.34–2.52	0.000	Fixed	0.0	0.926
	OA	1.29	1.13–1.48	0.000	Fixed	38.4	0.065
	Ethnicity						
	Asian	1.49	1.25–1.78	0.000	Fixed	15.5	0.300
	Caucasian	1.27	1.07–1.51	0.006	Fixed	33.8	0.120
Dominant model	Overall	1.56	1.39–1.75	0.000	Fixed	40.5	0.026
	Disease						
	IDD	2.11	1.57–2.86	0.000	Fixed	0.0	0.569
	OA	1.46	1.29–1.66	0.000	Fixed	46.2	0.026
	Ethnicity						
	Asian	1.82	1.54–2.16	0.000	Fixed	47.8	0.045
	Caucasian	1.35	1.15–1.58	0.000	Fixed	14.9	0.299
Recessive model	Overall	1.39	1.20–1.60	0.000	Random	73.3	0.000
	Disease						
	IDD	1.44	1.02–2.04	0.037	Random	71.4	0.002
	OA	1.35	1.16–1.59	0.000	Random	74.0	0.000
	Ethnicity						
	Asian	1.55	1.25–1.92	0.000	Random	83.2	0.000
	Caucasian	1.21	1.03–1.42	0.019	Fixed	36.2	0.101

*IDD* intervertebral disc degeneration, *OA* osteoarthritis, *OR* odds ratio, *CI* confidence interval

### Subgroup analysis

Because of heterogeneity, we conducted stratified analyses based on different disease types and ethnicity. Stratified analyses based on disease type revealed a significant relationship between the GDF5 rs143383 polymorphism and increased risk of IDD under all genetic models studied (allele model: OR = 1.45, 95% CI 1.13–1.86, *P* = 0.004; homozygote model: OR = 2.33, 95%CI 1.55–3.51, *P* = 0.000; heterozygote model: OR = 1.84, 95%CI 1.34–2.52, *P* = 0.000; dominant model: OR = 2.11, 95%CI 1.57–2.86, *P* = 0.000; recessive model: OR = 1.44, 95%CI 1.02–2.04, *P* = 0.037). Additionally, rs143383 polymorphism was related with increased OA risk in all genetic models (allele model: OR = 1.28, 95% CI 1.28 1.14–1.44, P = 0.000; homozygote model: OR = 1.67, 95%CI 1.36–2.05, *P* = 0.000; heterozygote model: OR = 1.29, 95%CI 1.13–1.48, *P* = 0.000; dominant model: OR = 1.46, 95%CI 1.29–1.66, *P* = 0.000; recessive model: OR = 1.35, 95%CI 1.16–1.59, *P* = 0.000) (Table 3).

When stratified with ethnicity, the outcomes revealed that this polymorphism was statistically related with increased risk of musculoskeletal degenerative diseases in Asian populations under all genetic models studied (allele model: OR = 1.49, 95% CI 1.25–1.77, *P* = 0.000; homozygote model: OR = 2.28, 95%CI 1.71–3.05, *P* = 0.000; heterozygote model: OR = 1.49, 95%CI 1.25–1.78, *P* = 0.000; dominant model: OR = 1.82, 95%CI 1.54–2.16, *P* = 0.000; recessive model: OR = 1.55, 95%CI 1.25–1.92, *P* = 0.000). In the Caucasian subgroup, a significant relationship between rs143383 polymorphism and increased risk of musculoskeletal degenerative diseases under all genetic models was also observed in our study (allele model: OR = 1.18, 95% CI 1.09–1.28, P = 0.000; homozygote model: OR = 1.46, 95%CI 1.23–1.74, P = 0.000; heterozygote model: OR = 1.27, 95%CI 1.07–1.51, *P* = 0.006; dominant model: OR = 1.35 95%CI 1.15–1.58, *P* = 0.000; recessive model: OR = 1.21, 95%CI 1.03–1.42, *P* = 0.019) (Table 3).

### Publication bias and sensitivity analysis

No obvious publication bias was shown in the funnel plot. In addition, there was no obvious publication bias according to Begg’s test (*P* = 0.338) and Egger’s test (*P* = 0.246). Therefore, we could exclude the possibility of publication bias. The sensitivity analysis revealed that the main outcomes of our study did not alter greatly when deleting studies one by one (Additional file 2).

## Discussion

Musculoskeletal degenerative diseases including IDD and OA are multifactorial diseases that bring about physical and functional limitations in patients. Various genetic risk factors may be responsible for the leading causes of IDD or OA [26, 27]. Previous studies have revealed that GDF5 polymorphism to be related with IDD, but with inconsistent results. Therefore, our study was made to assess the association between GDF5 rs143383 polymorphism and the susceptibility of IDD and OA. 15 articles with 915 patients and 12,252 controls were in our study. Eligible articles contained three studies in seven independent populations about IDD risk, and twelve studies assessed outcomes of GDF5 rs143383 on the risk of OA in fifteen independent populations.

GDF5 (+ 104 T/C; rs143383) is supposed to bring out a reduced transcription activity [28]. Our study revealed that GDF5 rs143383 polymorphism was significantly related with susceptibility to musculoskeletal degenerative diseases under all genetic models studied. Stratified analyses based on disease type showed a significant relationship between GDF5 rs143383 T allele and increased risk of IDD and OA. When stratified with ethnicity, the outcomes revealed that GDF5 rs143383 was statistically related with susceptibility to musculoskeletal degenerative diseases in both Asians and Caucasians.

Relatively obvious heterogeneities existed under all five genetic models in our study. With the aim of detecting the source of heterogeneity, we conducted sensitivity analysis and found that none articles altered the pooled OR significantly. Furthermore, we predicted that disease type and ethnicity may account for the heterogeneity and stratified analyses were then conducted. Neither the Egger test nor the Begg’s funnel plot revealed obvious publication bias for the IDD or OA risk related with GDF5 polymorphism. Even though the outcomes are reliable, additional studies are warranted to further confirm the findings.

Taken all these data in consideration, our study has several strengths. First, we utilized a comprehensive search method with well-defined inclusion and exclusion criteria. Second, two investigators accessed the eligibility of articles and selected related data separately. Third, we assessed the quality of included studies by well-defined criteria and the scores here were high. Finally, stratified analyses based on disease type and ethnicity were conducted to get a generalized conclusion.

Whereas, several limitations still existed in our study. First, the sample sizes in our study are relatively limited, which might bring about the insufficiency of statistical power. Second, the majority of articles included merely assessed the relationship between the gene polymorphism with IDD or OA risk, and more precise OR adjusted for other covariates such as age, sex, and environmental factors were not accessible. Finally, we concluded merely one representative SNP and articles including other GDF5 polymorphisms are needed.

## Conclusion

Our study demonstrated that GDF5 rs143383 polymorphism was significantly related with susceptibility to musculoskeletal degenerative. More studies are warranted to investigate the value of GDF5 polymorphisms and variations in other genes for years to come.

## Additional files

Additional file 1: **Table S1.** Quality assessment of eligible studies (Newcastle-Ottawa Scale). **Table S2.** Definitions of disease or inclusions and exclusions in eligible studies. (DOCX 26 kb)

Additional file 2: **Figure S2.** Funnel plot for GDF5 polymorphism in musculoskeletal degenerative diseases. **Figure S3.** Begg’s funnel plot for GDF5 polymorphism in musculoskeletal degenerative diseases. **Figure S4.** Egger’s funnel plot for GDF5 polymorphism in musculoskeletal degenerative diseases. **Figure S5.** Sensitivity analysis for GDF5 polymorphism in musculoskeletal degenerative diseases. (DOCX 864 kb)
