# Supplementary material for: Association between GDF5 rs143383 genetic polymorphism and musculoskeletal degenerative diseases susceptibility: a meta-analysis
Source: BMC Med Genet. 2018 Sep 14;19:169. doi: 10.1186/s12881-018-0685-7 (PMC6137727; doi:10.1186/s12881-018-0685-7)
Supplement: Supplementary file 1 — Table S1. Quality assessment of eligible studies (Newcastle-Ottawa Scale). Table S2. Definitions of disease or inclusions and exclusions in eligible studies. (DOCX 26 kb) [file 12881_2018_685_MOESM1_ESM.docx]

**Additional file 1**

**Supplementary Table 1** Quality assessment of eligible studies (Newcastle-Ottawa Scale)

| **Study** | **Selection** | | | **Comparability** | | | **Outcome** | | **Total** |
| --- | --- | --- | --- | --- | --- | --- | --- | --- | --- |
|  | **Adequacy of case definition** | **Number**  **of case** | **Representativeness of the cases** | **Ascertainment of**  **exposure** | **Ascertainment of**  **detection method** | **Ascertainment of cut-off** | **Assessment of outcome** | **Adequate**  **follow up** |  |
| Mu J | 1 | 1 | 1 | 1 | 1 | 1 | 1 | 1 | 8 |
| Mu J | 1 | 1 | 1 | 1 | 1 | 1 | 1 | 1 | 8 |
| Williams FMK ^a^ | 1 | 1 | 1 | 1 | 1 | 1 | 1 | 0 | 7 |
| Williams FMK ^b^ | 1 | 1 | 1 | 1 | 1 | 1 | 1 | 0 | 7 |
| Williams FMK ^c^ | 1 | 1 | 1 | 1 | 1 | 1 | 1 | 0 | 7 |
| Williams FMK ^d^ | 1 | 1 | 1 | 1 | 1 | 1 | 1 | 0 | 7 |
| Williams FMK ^e^ | 1 | 1 | 1 | 1 | 1 | 1 | 1 | 0 | 7 |
| Tülüce Y | 1 | 1 | 1 | 1 | 1 | 1 | 1 | 0 | 7 |
| Abd Elazeem MI | 1 | 1 | 1 | 1 | 1 | 1 | 1 | 0 | 7 |
| Sabah-Ozcan S | 1 | 1 | 1 | 1 | 1 | 1 | 1 | 1 | 8 |
| Xiao JL | 1 | 1 | 1 | 1 | 1 | 1 | 1 | 0 | 7 |
| Mishra A | 1 | 1 | 1 | 1 | 1 | 1 | 1 | 1 | 8 |
| Tawonsawatruk T | 1 | 1 | 1 | 1 | 1 | 1 | 1 | 0 | 7 |
| Cao Z | 1 | 1 | 1 | 1 | 1 | 1 | 1 | 0 | 7 |
| Valdes AM | 1 | 1 | 1 | 1 | 1 | 1 | 1 | 0 | 7 |
| Tsezou A | 1 | 1 | 1 | 1 | 1 | 1 | 1 | 0 | 7 |
| Miyamoto Y ^a^ | 1 | 1 | 1 | 1 | 1 | 1 | 1 | 0 | 7 |
| Miyamoto Y ^b^ | 1 | 1 | 1 | 1 | 1 | 1 | 1 | 1 | 8 |
| Miyamoto Y ^c^ | 1 | 1 | 1 | 1 | 1 | 1 | 1 | 0 | 7 |
| Southam L ^a^ | 1 | 1 | 1 | 1 | 1 | 1 | 1 | 0 | 7 |
| Southam L ^b^ | 1 | 1 | 1 | 1 | 1 | 1 | 1 | 0 | 7 |
| Shin MH | 1 | 1 | 1 | 1 | 1 | 1 | 1 | 0 | 7 |

**Supplementary Table 2:** Definitions of disease or inclusions and exclusions in eligible studies.

| **Study** | **Citation** | **Disease** | **Definitions of disease or inclusion** | **Exclusion** |
| --- | --- | --- | --- | --- |
| Mu J | [17] | LDH | Patients had a history of unilateral pain radiating from the back along the femoral or sciatic nerve to the corresponding dermatome of the nerve root for more than 1 month and positive MRI findings for LDH. The symptoms should be in accordance with compression of the lumbar disc. | OA, previous fractures of the spine, lumbar spinal stenosis, malignancies involving the spine and poliomyelitis were excluded from all the subjects. |
| Mu J | [18] | LBP | Patients had a history of LBP with or without radiculopathy for more than 2 weeks. | Subjects were excluded if they were unable to meet these criteria. Fractures of the spine, malignancies involving the spine, obvious trauma, and a history of LBP prior to enlistment were exclusion criteria. |
| Williams FMK | [19] | LDD | Subjects were considered to have LDD if, considering the uppermost 4 lumbar discs, either narrowing was present at ≥2 levels, osteophytes were present at ≥2 levels, or there was a combination of narrowing and osteophytes. | Not available |
| Tülüce Y | [20] | OA | Clinical and radiological diagnostic criteria were based on the American College of Rheumatology criteria and the Kellgren and Lawrence scores. | Patients with rheumatoid arthritis, with chondrodysplasia, infection-induced OA and post-rheumatic OA were excluded from the study. |
| Abd Elazeem MI | [21] | Primary knee OA | Patients were diagnosed by a rheumatologist according to the American College of Rheumatology classification for knee OA. | Patients with a history of knee or hip surgery, secondary OA, other rheumatic diseases, and knee trauma were excluded from the study. |
| Sabah-Ozcan S | [22] | Knee OA | Clinical and radiological diagnostic criteria were based on the American College of Rheumatology criteria and the Kellgren and Lawrence scores, respectively. | Exclusion criteria were age less than 50 years; history of bone fracture in the knee; diagnosis or treatment history of inflammatory arthritis (e.g., rheumatoid arthritis); or any hematologic disorder. |
| Xiao JL | [23] | Temporomandibular  joint OA | Diagnosis was based on Research Diagnostic Criteria for Temporomandibular Disorders (RDC/TMD) and on cone-beam computed tomography, which showed erosion, sclerosis, osteophytes, flattening and subchondral cysts of the condyles. | According to RDC/TMD, patients with maxillofacial trauma history, systemic endocrine disturbances, multiple OA, rheumatism, rheumatoid arthritis or other connective tissue diseases were also excluded based on their medical history and related laboratory examinations. |
| Mishra A | [5] | Knee OA | Patients fulfilled American College of Rheumatology clinical and radiographic criteria for knee OA. | Not available |
| Tawonsawatruk T | [26] | Knee OA | Diagnosis of knee OA was based on the American College of Rheumatology criteria. Moreover, standard weightbearing antero-posterior and lateral view of knee radiographs were taken to confirm the diagnosis of OA by Kellgren and Lawrence scores. | Not available |
| Cao Z | [27] | Knee OA | Clinical and radiological diagnostic criteria were based on the American College of Rheumatology criteria and the Kellgren and Lawrence scores. | Not available |
| Valdes AM | [28] | Knee OA | The Kellgren and Lawrence (KL) grade was scored for the tibio-femoral compartment of each knee. Radiographic knee OA was defined as a KL score ≥2 of one or both joints. | Not available |
| Tsezou A | [29] | Knee OA | Patients had a Kellgren Lawrence score ≥2 and were randomly selected. The radiographs were assessed by two independent expert observers who were blinded to all data for the patients. | Patients with rheumatoid arthritis and other autoimmune diseases as well as chondrodysplasias, infection-induced OA, and posttraumatic OA were not included in the study. |
| Miyamoto Y | [30] | Knee OA | All affected individuals showed symptoms (pain, limp and limitation of the joint movement) and radiographic signs (narrowing of the joint space and formation of osteophytes) of OA. Clinical and radiological diagnostic criteria were based on the American College of Rheumatology criteria and the Kellgren and Lawrence scores, respectively. | Not available |
| Southam L | [31] | Knee OA | Patients fulfilled American College of Rheumatology classification criteria. | Not available |
| Shin MH | [32] | Knee OA | The radiographs were read by two examiners who were blinded to the clinical information, and they used an atlas of radiographic features to obtain a global Kellgren/Lawrence (K/L) score (0-4 scale). Radiographic OA was defined as a K/L score of ≥2. | Inflammatory arthritis (rheumatoid, polyarthritic or autoimmune disease), posttraumatic or post-septic arthritis were excluded |

LDD, lumbar disc degeneration; LDH, lumbar disc herniation; LBP, low-back pain; OA, osteoarthritis.
