# Supplementary material for: Association between GDF5 rs143383 genetic polymorphism and musculoskeletal degenerative diseases susceptibility: a meta-analysis
Source: BMC Med Genet. 2018 Sep 14;19:169. doi: 10.1186/s12881-018-0685-7 (PMC6137727; doi:10.1186/s12881-018-0685-7)
Supplement: Supplementary file 2 — Figure S2. Funnel plot for GDF5 polymorphism in musculoskeletal degenerative diseases. Figure S3. Begg’s funnel plot for GDF5 polymorphism in musculoskeletal degenerative diseases. Figure S4. Egger’s funnel plot for GDF5 polymorphism in musculoskeletal degenerative diseases. Figure S5. Sensitivity analysis for GDF5 polymorphism in musculoskeletal degenerative diseases. (DOCX 864 kb) [file 12881_2018_685_MOESM2_ESM.docx]

**Additional file 2**

**
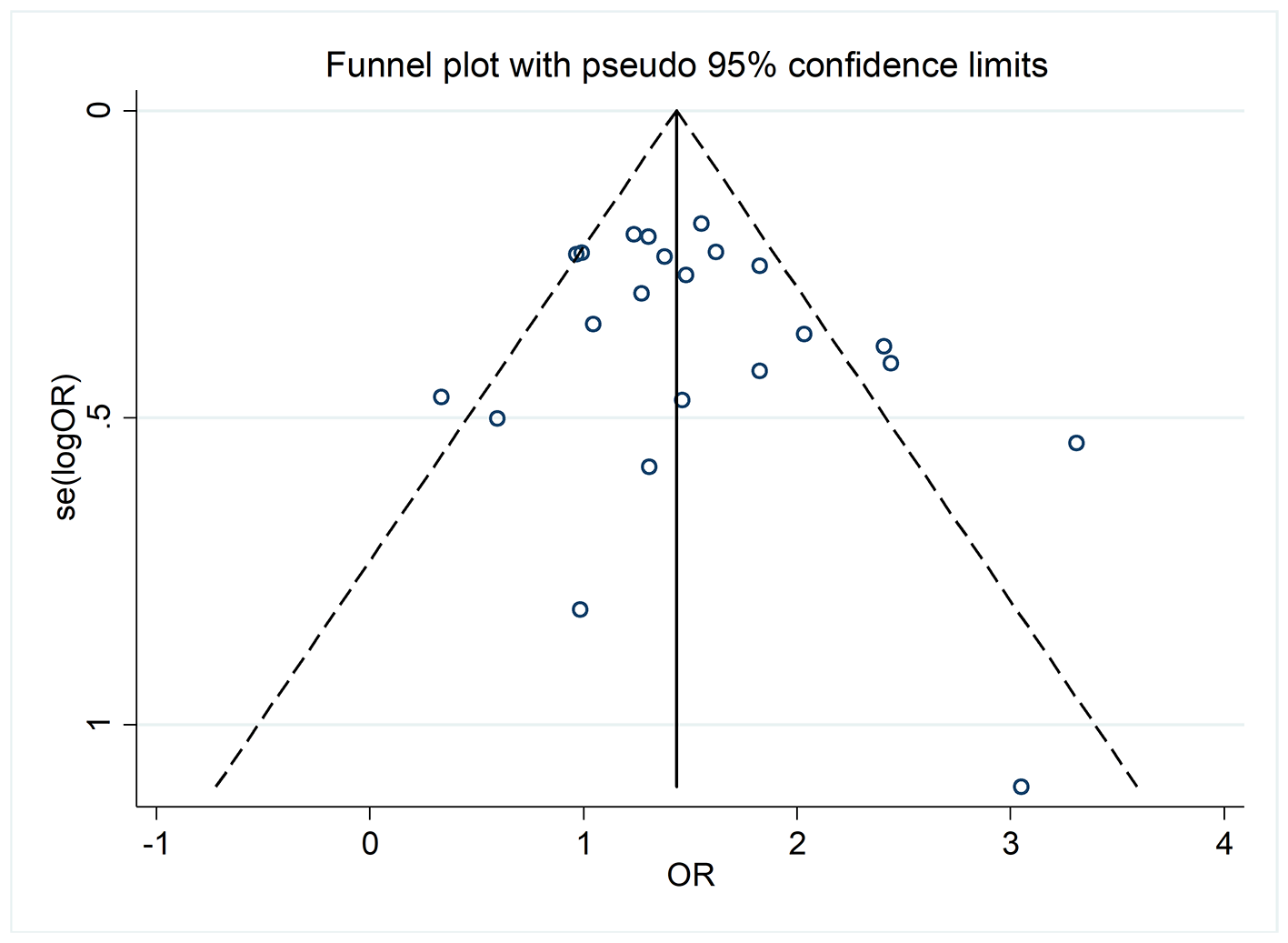
**

**Supplementary Figure 2:** Funnel plot for GDF5 polymorphism in musculoskeletal degenerative diseases.


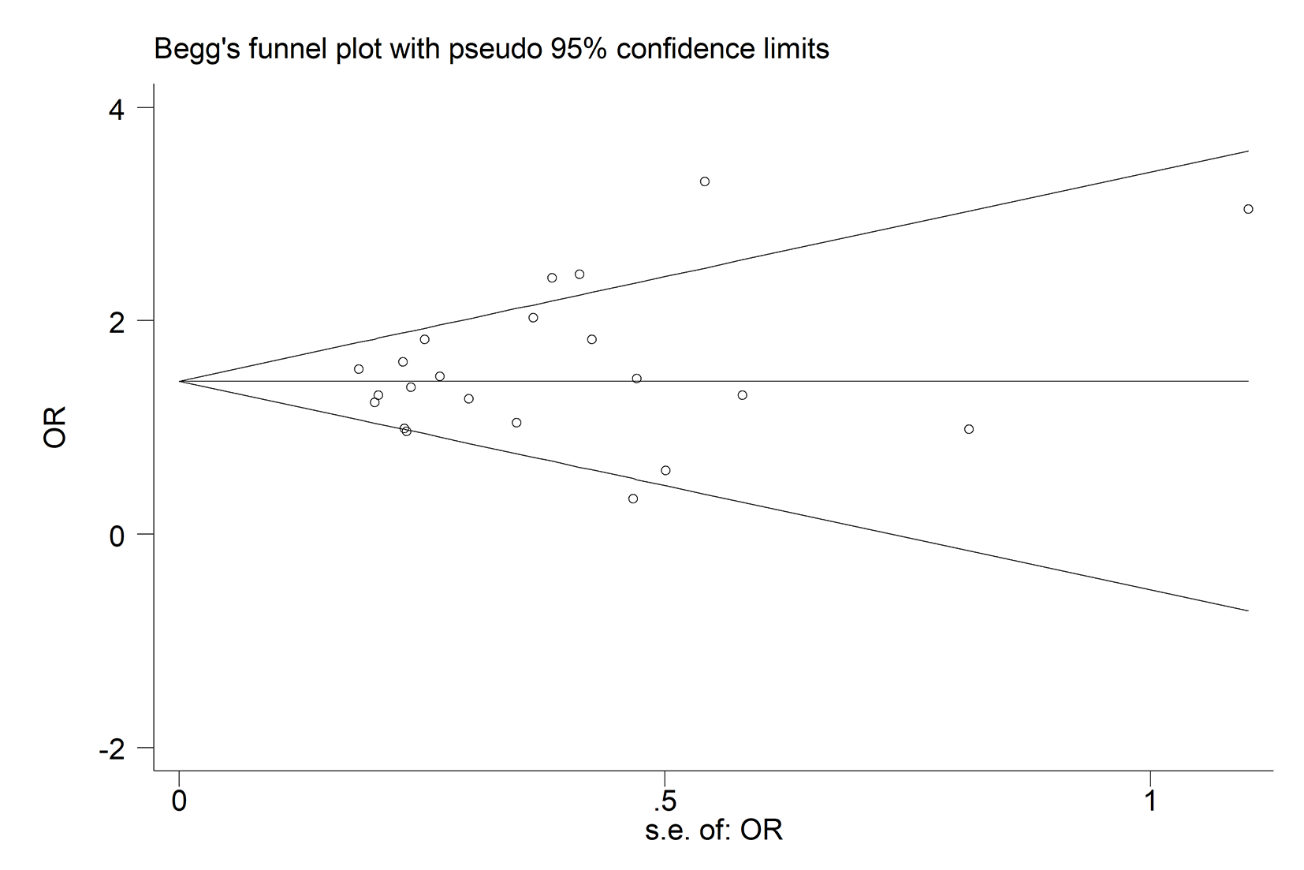


**Supplementary Figure 3:** Begg’s funnel plot for GDF5 polymorphism in musculoskeletal degenerative diseases.


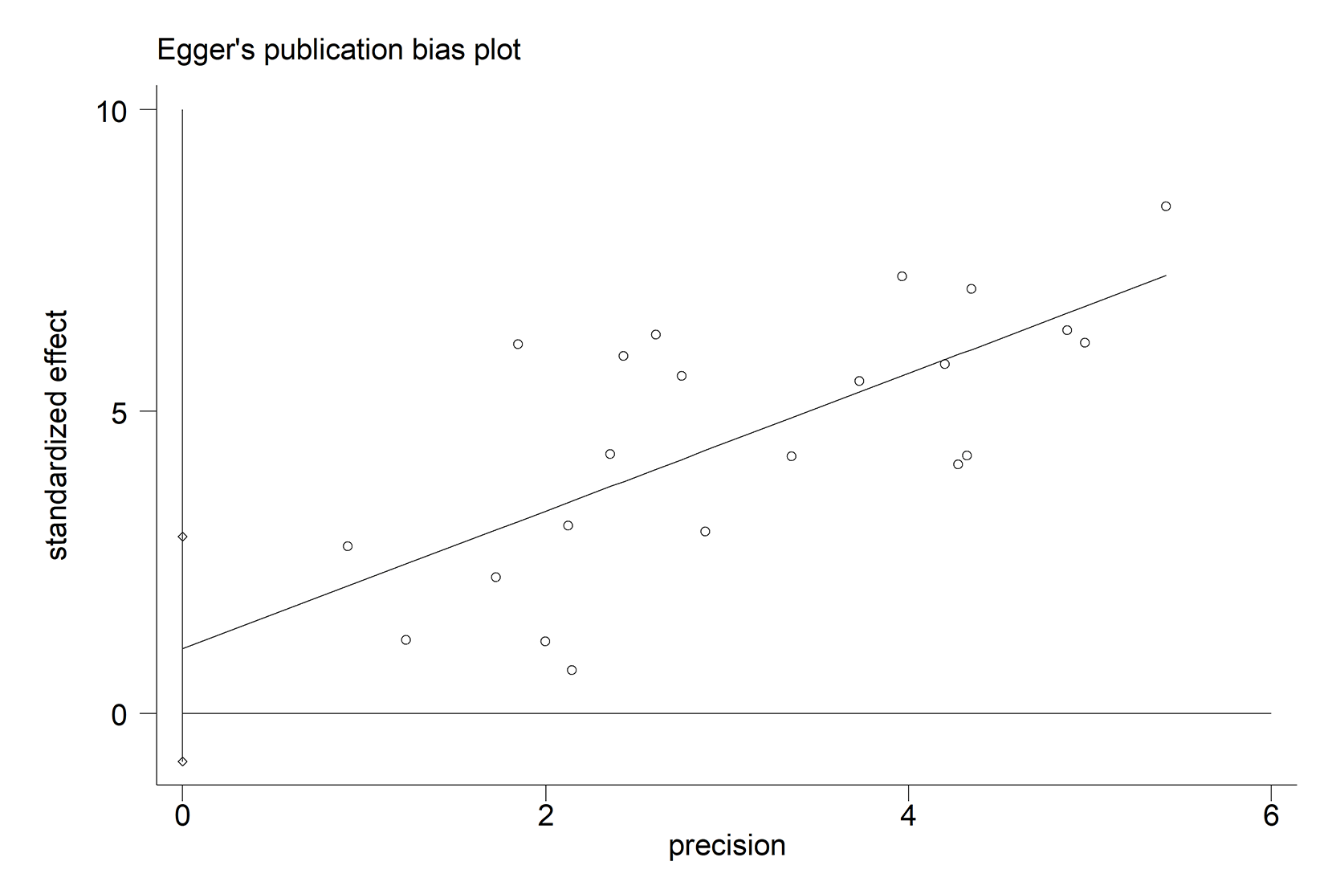


**Supplementary Figure 4:** Egger’s funnel plot for GDF5 polymorphism in musculoskeletal degenerative diseases.


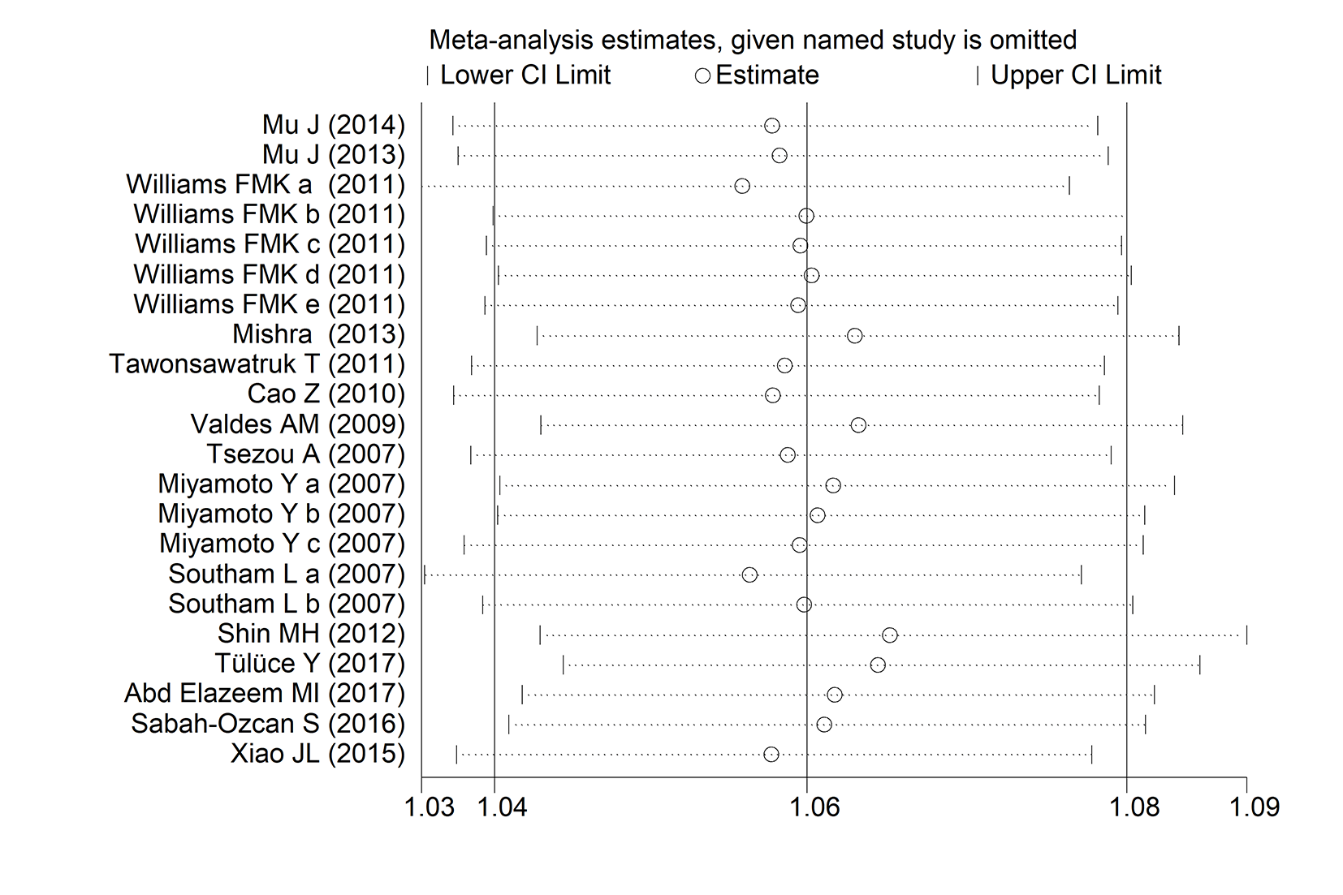


**Supplementary Figure 5:** Sensitivity analysis for GDF5 polymorphism in musculoskeletal degenerative diseases.
